# Interferon Gamma Alters the Phenotype of Rat Thymic Epithelial Cells in Culture and Increases Interleukin-6 Production

**DOI:** 10.1155/1992/60719

**Published:** 1992

**Authors:** Miodrag Čolić, Nada Pejnović, Milena Kataranovski, Ljiljana Popović, Sonja GašIć, Aleksandar Dujić

**Affiliations:** Institute of Medical Research Military Medical Academy Belgrade Serbia and Montenegro

**Keywords:** Thymic epithelial cells, culture, cytokines, monoclonal antibodies, electron microscopy

## Abstract

Rat thymic epithelial cells (TEC) in long-term culture were characterized by anticytokeratin monoclonal antibodies (mAbs) and electron microscopy. Phenotypic analysis performed by a large panel of mAbs showed that the highest percentage of these cells was of the subcapsular/medullary type. Recombinant rat interferon (IFN)-gamma up-regulated class-I and class-II MHC expression by TEC in culture as confirmed by immunohistochemistry and flow cytometry, but did not significantly alter other cell markers. TEC supernatants of IFN-gamma- treated cultures showed higher interleukin-6 (IL-6) activity, compared to the control, as determined by proliferation of the IL-6-sensitive B9-cell line. Increased IL-6 activity was probably not a consequence of increased TEC number in IFN-gammatreated cultures because IFN did not significantly stimulate TEC proliferation *in vitro*. In contrast, IL-6 significantly stimulated TEC proliferation, indicating that this cytokine is not only a regulatory molecule for T-cell proliferation, but could also be an autocrine growth factor for thymic epithelium.


*Developmental Immunology*, 1992, Vol. 2, pp. 151–160
Reprints available directly from the publisher
Photocopying permitted by license only

© 1992 Harwood Academic Publishers GmbH
Printed in the United Kingdom

### Interferon Gamma Alters the Phenotype of Rat Thymic Epithelial Cells in Culture and Increases Interleukin-6 Production

MIODRAG ČOLIĆ,* NADA PEJNOVIĆ, MILENA KATARANOVSKI, LJILJANA POPOVIĆ, SONJA GAŠIĆ, and ALEKSANDAR DUJIĆ

*Institute of Medical Research, Military Medical Academy, Belgrade, Yugoslavia*

Rat thymic epithelial cells (TEC) in long-term culture were characterized by anticytokeratin monoclonal antibodies (mAbs) and electron microscopy. Phenotypic analysis performed by a large panel of mAbs showed that the highest percentage of these cells was of the subcapsular/medullary type.

Recombinant rat interferon (IFN)-gamma up-regulated class-I and class-II MHC expression by TEC in culture as confirmed by immunohistochemistry and flow cytometry, but did not significantly alter other cell markers. TEC supernatants of IFN-gamma-treated cultures showed higher interleukin-6 (IL-6) activity, compared to the control, as determined by proliferation of the IL-6-sensitive B9-cell line. Increased IL-6 activity was probably not a consequence of increased TEC number in IFN-gamma-treated cultures because IFN did not significantly stimulate TEC proliferation *in vitro*. In contrast, IL-6 significantly stimulated TEC proliferation, indicating that this cytokine is not only a regulatory molecule for T-cell proliferation, but could also be an autocrine growth factor for thymic epithelium.

KEYWORDS: Thymic epithelial cells, culture, cytokines, monoclonal antibodies, electron microscopy.

### INTRODUCTION

The importance of the thymic microenvironment, which is composed of epithelial cells, macrophage/dendritic cells, and fibrous stroma (Lobach and Haynes, 1988; van Ewijk, 1988), in T-cell development has been well established (Lobach and Haynes, 1988; Sprent et al., 1988). However, the role of individual components in thymocyte positioning, differentiation, and proliferation as well as development of a self-MHC restricted T-cell repertoire has not been fully elucidated. In order to study the relationship between thymic-microenvironmental cells and thymocytes, several investigators have used a more direct approach involving *in vitro* cell culture assays. However, many difficulties in maintaining pure populations, especially thymic epithelial cells (TEC) were reported (Janson and Janeway, 1984; Sun et al., 1984; Osculati et al., 1988). TEC growth has been promoted with low calcium, serum-free medium (Piltch et al., 1988), medium supplemented with D-valine (Small et al., 1984; Nieburgs et al., 1985; Farr et al., 1986), extracellular matrix (Eshel et al., 1990), or irradiated fibroblasts as filler cells (Farr et al., 1986; Le et al., 1987). Sometimes, results were not satisfactory.

We succeeded in successfully cultivating pure rat TEC population using RPMI-1640 medium with addition of 15% fetal calf serum (FCS), insulin, dexamethasone, epidermal growth factor (EGF) and poly-L-lysine (PLL) as an adhesive matrix. One long-term culture passaged for more than 8 months was used in these experiments to study the effect of recombinant rat interferon (INF)-gamma on the phenotype and cytokine production by TEC. To our knowledge, it is the first report showing that INF-gamma stimulates IL-6 production by thymic epithelium *in vitro*.

*Corresponding author.

### RESULTS

### Characterization of Rat TEC in Culture by Immunohistochemistry and Electron Microscopy

A rat TEC culture successfully grown on PLL-coated flasks and propagated for more than 8 months was used in these experiments. The epithelial nature of these cells was confirmed using K.8.13 mAb recognizing various CK polypeptides, a panepithelial marker of rat TEC *in situ* (Čolić et al., 1989). TEC were 99% K.8.13+ (Table 2). Figure 1 shows a monolayer of these cells in which typical filamentous intracytoplasmic labeling is seen using K.8.13 mAb and indirect immunofluorescence.

We also used electron microscopy and found ultrastructural characteristics of epithelial cells such as tonofilaments and desmosomes (Fig. 2).

### Effect of Recombinant IFN-Gamma on TEC Phenotype

Rat TEC from long-term culture described above were treated with 50 IU/mL or 200 IU/mL of recombinant rat IFN-gamma. After 48 hr, cells were phenotypically analyzed. Cytospins were stained with a large panel of mAbs (Table 1), including those reactive with CK polypeptides, R-MC (Čolić et al., 1988), or HIS (Kampinga et al., 1987) series. In addition, mAbs detecting rat class-I and class-II MHC molecules were also used.

Table 2 shows that both in control and IFN-gamma-treated cultures most cells were stained with mAbs recognizing subcapsular/medullary epithelium *in situ*, such as CK 19, K.8.12, R-MC 18, R-MC 19, and HIS 39. However, no significant difference was observed in IFN-gamma-treated cultures compared to controls.

The greatest differences were obtained by staining with anticlass-I (OX-18) and class-II MHC (OX-6 and OX-17) mAbs. Control cultures were almost all both IA (OX-6) and IE (OX-17) negative. Approximately 60% of the cells were moderately OX-18 positive. IFN-gamma caused dose-dependent up-regulation of class-I and class-II MHC expression (Table 2). This observation was confirmed using flow cytometry (Fig. 3).

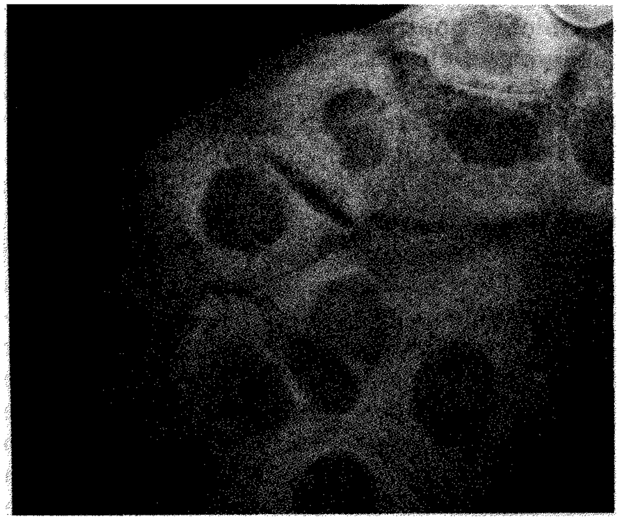

FIGURE 1. A monolayer of rat TEC culture stained with K.8.13 mAb using streptavidin-biotin immunofluorescence. Note intracytoplasmic filamentous staining. Magnification: × 520.

### IFN-Gamma Stimulates IL-6 Production by Rat TEC

Confluent TEC cultures were treated for 48 hr with different doses of IFN-gamma (25, 50, 100, and 200 IU/mL). Supernatants were collected and tested for IL-6 activity using the IL-6-sensitive B9-cell line. Figure 4 shows that IFN-gamma-stimulated IL-6 production by these cells in a dose-dependent manner.

### Effect of Cytokines on TEC Proliferation *In Vitro*

To address the question whether increased production of IL-6 is a consequence of increased TEC number in IFN-gamma-treated cultures, we studied the effect of IFN-gamma on TEC proliferation by $^{3}$H thymidine uptake. Figure 5 shows that none of the different IFN-gamma doses significantly altered the TEC proliferation compared to the control. In contrast, TEC cultures treated with recombinant IL-6 showed significantly higher proliferation at some doses.

### DISCUSSION

Cultivation of TEC *in vitro* is of great importance in studying their effect of T-cell development.

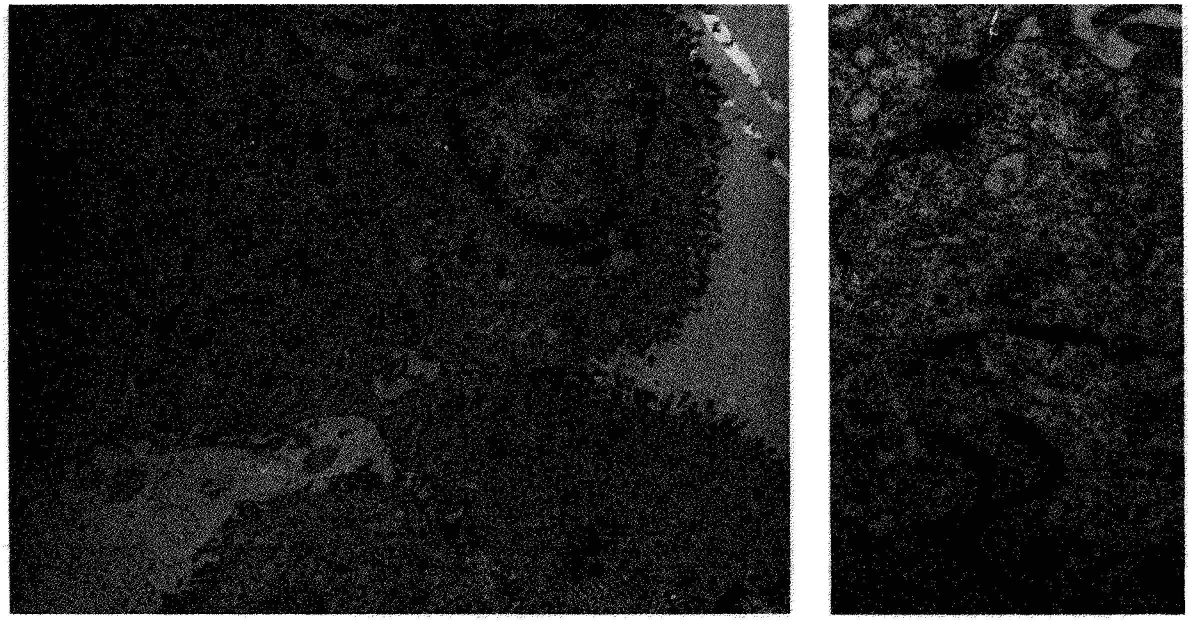

FIGURE 2. (a) Electron-microscopic analysis of rat TEC culture. Magnification: ×11,200. (b) Ultrastructural characteristics of epithelial cells such as tonofilaments (t) and desmosomes (d) are shown separately. Magnification: ×19,400.

TABLE 1
Immunoreactivity of mAbs on Adult Rat Thymus

| MAbs | Staining pattern | Reference |
|---|---|---|
| K.8.13 | Panepithelium | Čolić et al., 1989 |
| CK 8 | Panepithelium | Čolić et al., 1989 |
| K.8.12 | Subcapsule/PV[a]; subset of medullary TEC | Čolić et al., 1989 |
| CK 18 | Cortical TEC; subset of medullary TEC | Čolić et al., 1989 |
| CK 19 | Subcapsule/PV[a]; subset of medullary TEC | Čolić et al., 1989 |
| KL1 | Subset of medullary TEC; HC[b] | Čolić et al., 1989 |
| R-MC 14 | Cortical TEC | Čolić et al., 1988 |
| R-MC 16 | Cortical TEC[c] | Čolić et al., 1988 |
| R-MC 17 | Cortical TEC; subset of medullary TEC | Čolić et al., 1988 |
| R-MC 18 | Subcapsule/PV[a]; most medullary TEC | Čolić et al., 1988 |
| R-MC 19 | Subcapsule/PV[a]; most medullary TEC | Čolić et al., 1988 |
| R-MC 20 | Subcapsule/PV[a]; most medullary TEC; some HC[b] | Čolić et al., 1988 |
| HIS 37 | Cortical TEC; subset of medullary TEC | Kampinga et al., 1987 |
| HIS 38 | Cortical TEC; subset of medullary TEC | Kampinga et al., 1987 |
| HIS 39 | Subcapsule/PV[a]; most medullary TEC | Kampinga et al., 1987 |
| OX-6 (anti-IA) | Panepithelium (cortex-strong; medulla-weak)[d] IDC, some macrophages, subset of thymocytes | |
| OX-17 (anti-IE) as OX-6[d] | | |
| OX-18 (anti-class I MHC) almost all cells in the thymus[d] | | |

[a]PV=perivascular epithelium.

[b]HC-Hassall's corpuscles.

[c]Rare medullary TEC are weakly stained.

[d]Reactivity on rat thymus tested in our laboratory.

However, many difficulties in obtaining pure TEC population have been reported (reviewed by Osculati et al., 1988) due to overgrowth of fibroblasts and contamination of cultures with other nonlymphoid cells.

In our experiments, we resolved this problem using selective components that promote TEC growth and suppress fibroblast proliferation such as EGF, dexamethasone, and insulin. These components have already been used for cultivation of human (Le et al., 1987), mouse (Small et al., 1984, 1989; Ehmann et al., 1986; Eshel et al.,

TABLE 2
Effect of IFN-Gamma on Phenotype of Rat TEC in Culture[a]

| mAbs | Control | | IFN-gamma, 50 IU/ml | | IFN-gamma, 200 IU/ml | |
|---|---|---|---|---|---|---|
| | % | Intensity | % | Intensity | % | Intensity |
| K.8.13 | 99 | ++[b] | 100 | ++ | 99 | ++ |
| CK 8 | 100 | ++ | 100 | ++ | 99 | ++ |
| K.8.12 | 82 | ++ | 80 | ++ | 84 | ++ |
| CK 18 | 0 | | 0 | | 0 | |
| CK 19 | 36 | ++ | 34 | ++ | 34 | ++ |
| KL 1 | 0 | | 1 | ±[c] | 0 | |
| R-MC 14 | 0 | | 0 | | 0 | |
| R-MC 16 | 1 | ± | 0 | | 1 | ± |
| R-MC 17 | 3 | ± | 4 | ± | 3 | ± |
| R-MC 18 | 79 | ++ | 81 | ++ | 78 | ++ |
| R-MC 19 | 66 | +[d] | 59 | + | 62 | + |
| R-MC 20 | 7 | + | 9 | + | 7 | + |
| HIS 37 | 4 | + | 2 | + | 3 | + |
| HIS 38 | 0 | | 0 | | 0 | |
| HIS 39 | 46 | + | 51 | + | 51 | + |
| OX-6 | 2 | ± | 67 | + | 84 | + |
| OX-17 | 1 | ± | 59 | ± or + | 82 | + |
| OX-18 | 59 | + | 93 | + or ++ | 99 | + or ++ |

[a]The percentages of positive cells are given on the basis of 200–400 analyzed cells on each cytospin preparation.

[b]++: strong labeling.

[c]±: weak labeling.

[d]+: moderate labeling.

1990), and rat (Piltch et al., 1988) thymic epithelium. The long-term cultivation of TEC was improved using PLL, an adhesive matrix widely used for cell and tissue adherence to glass and plastics. After several passages, contaminants completely disappeared and the only cells in culture were epithelial cells. Their epithelial nature was confirmed using anticytokeratin mAbs. The TEC culture used in this study was 99–100% positive with K.8.13 and CK 8 mAbs. We have previously shown that these mAbs are panepithelial markers for rat TEC *in situ* (Čolić et al., 1989). In addition, we performed electron microscopy and found ultrastructural markers of epithelial cells such as tonofilaments and desmosomes.

Based on the reactivity of TEC used in this study with a large panel of mAbs detecting various TEC subsets *in situ* (Kampinga et al., 1987; Čolić et al., 1988, 1989), it was concluded that this culture probably consisted of phenotypically heterogeneous cells, but most of them were of subcapsular/medullary type. It is interesting that many established TEC lines are also of medullary origin (Potworowski et al., 1986; Piltch et al., 1988; Farr et al., 1989), suggesting that this cell subset grows more easily *in vitro*.

The main aspect of this work evaluated the effect of IFN-gamma on TEC in culture. It is well known that IFN-gamma, produced by activated lymphocytes, is a cytokine involved in many immunological functions. Its effect on membrane expression of class-II MHC molecules (Berrih et al., 1985), ICAM-1 (Renkonen et al., 1990), and other antigens is well-documented. Previous studies on the human thymus demonstrated that TEC in culture lost class-II MHC expression, but this could be up-regulated by IFN-gamma (Berrih et al., 1985). Similar results have been published on mouse TEC from long-term cultures (Farr et al., 1989) or cloned mouse TEC from long-term cultures (Farr et al., 1989) or cloned mouse TEC lines (Ransom et al., 1987). We also demonstrated in this work that rat recombinant IFN-gamma up-regulated class-I and class-II MHC antigen expression by rat TEC, indicating again that this cytokine has an important physiological role in the thymus. This hypothesis was confirmed in the experiments showing that activated thymocytes produce IFN-gamma (Ransom et al., 1987). However, it is not clear which subsets of immature or mature thymocytes are IFN-gamma-secreting cells. Furthermore, experiments performed by Ransom et al. (1987) clearly showed that mouse TEC cultures are capable of presenting antigen following induction with IFN-gamma. Antigen-presenting function was Ia-restricted and directly correlated with TEC Ia expression after addition of IFN-gamma.

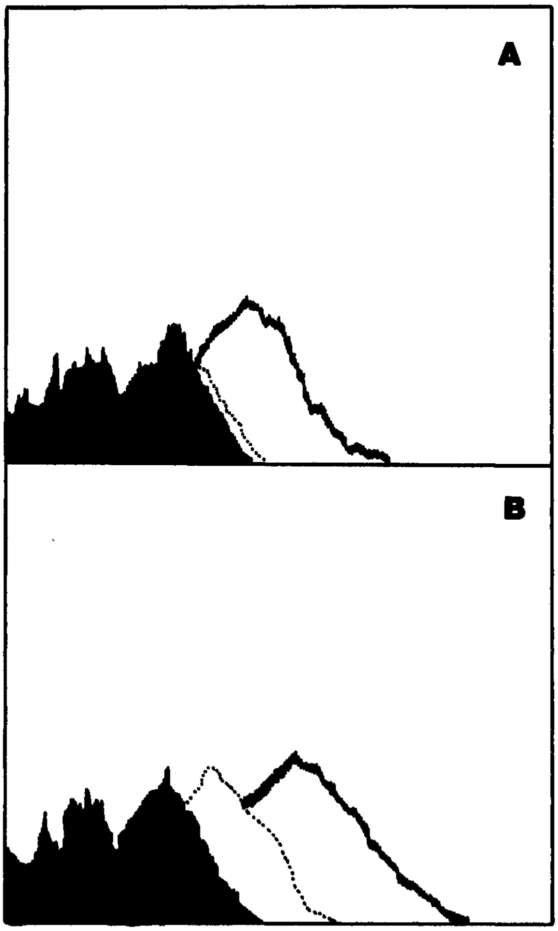

FIGURE 3. Effect of INF-gåmma on class-I and class-II MHC expression by rat TEC in culture. (A) staining with OX-6 mAb (anti-class II); (B) staining with OX-18 mAb (anti-class I). In each sample, 5000 TEC were analyzed. Black area: negative controls using an IgG1 irrelevant mouse mAb. Dotted lines: unstimulated (control) TEC. Hashed lines: TEC stimulated with 200 IU/ml of rat IFN-gamma. X axis=log fluorescence. Y-axis=cell number.

We also found that IFN-gamma stimulated IL-6 production by TEC in culture. This effect was dose-dependent and independent of TEC proliferation because IFN-gamma did not significantly alter TEC growth. To our knowledge, no such data have been published, although it was recently demonstrated that IFN-gamma induced IL-6 production by endothelial cells (Howells et al., 1991).

It is known that TEC secrete various cytokines, including IL-1, hematopoietic growth factors (GM-CSF, M-CSF, G-CSF), leukemia inhibitory factor, thymic hormones, prostaglandins, and other biological active molecules (reviewed by Haynes, 1990). It was recently shown that human (Le et al., 1990) and mouse (Murray et al., 1989) TEC also secrete IL-6. In our previous paper (Čolić et al., manuscript submitted), we tested supernatants from different primary and long-term rat TEC cultures and found in all of them constitutive presence of both IL-1 and IL-6. The concentration of IL-6 was determined by the B9-cell assay and recombinant human IL-6 as a standard. IL-6 concentration ranged from 220–2300 IU/ml. We also found that TEC in culture and *in situ* were reactive with antihuman IL-6 antibody. However, this antibody completely inhibited human IL-6 activity in the assay, but did not completely abrogate IL-6 activity in TEC supernatants.

Similar data were reported for another anti-IL6 polyclonal antibody that was not effective in complete neutralization of IL-6 in biological samples (see Genzyme catalogue, 1990). This finding could be explained by incomplete cross-reactivity of the antibody with rat IL-6 or that B9 cells could also respond to certain novel cytokines. For example, it has been recently demonstrated that certain plasmocytoma cell lines used for detection of IL-6 also respond to IL-11 (Paul et al., 1990). For this reason, the IL-6 activity is given in cpm in the experiments presented here.

The significance of IL-6 in thymocyte proliferation is well-documented. It enhances the proliferation of thymocytes in the presence of IL-2 (van Snick, 1990) and IL-4 (Suda et al., 1990) and also induces IL-2 and IL-2 receptor expression (van Snick, 1990; Sehgal, 1990). Our experiments clearly showed that IL-6 stimulated TEC proliferation, indicating tht it could also be an autocrine growth factor for thymic epithelium. No such finding was reported, although the effect of IL-6 on the growth of other nonlymphoid cells such as fibroblasts (Kohase et al., 1986), mesangial cells, and keratinocytes (Sehgal, 1990) has already been published. It was reported that IL-1 stimulates the proliferation of murine TEC *in vitro* and changes their morphology from cuboidal to fibroblastoid form (Galy et al., 1989). We also demonstrated similar proliferative effect of IL-1 on rat TEC (unpublished data) and are now in the process of testing whether IL-1-induced TEC proliferation is actually mediated by IL-6.

Another possible function of IL-6 related to the thymic microenvironment could be its effect on thymic macrophages. Knowing that IL-6 affects

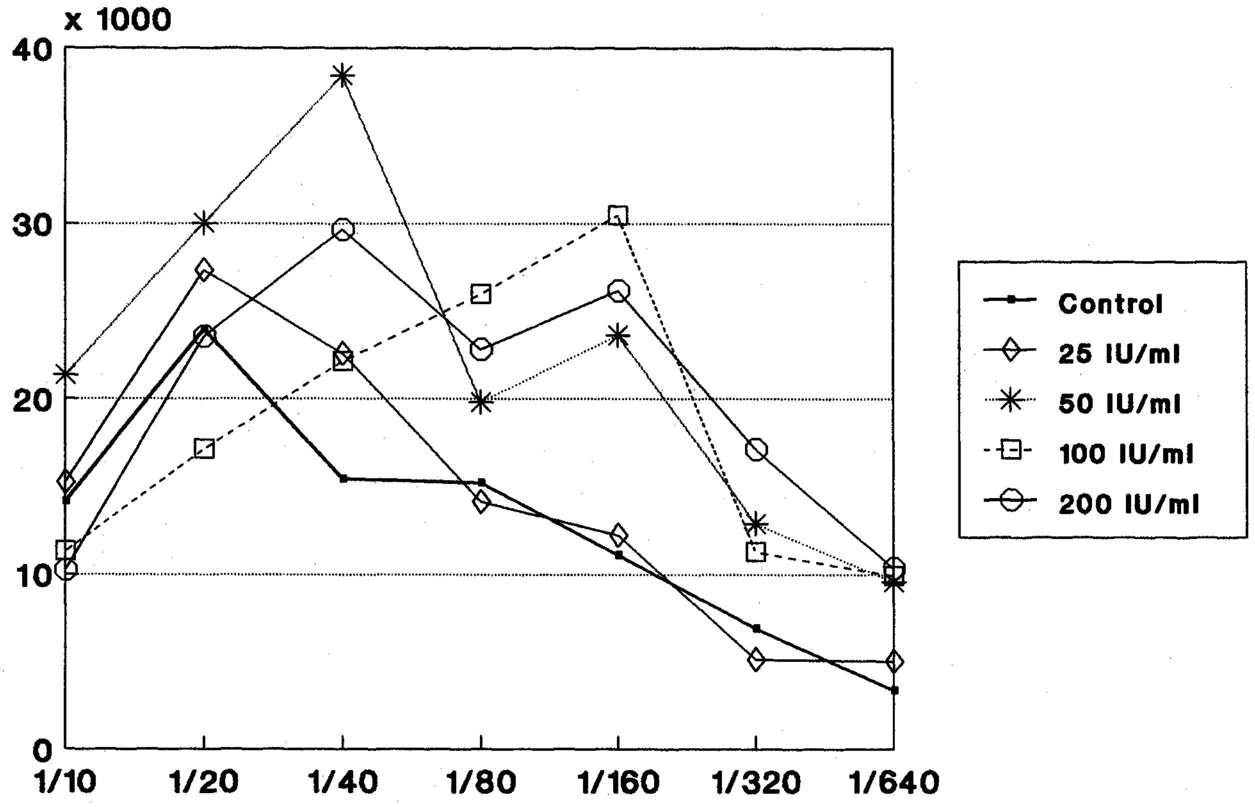

FIGURE 4. Effect of IFN-gamma on IL-6 production by rat TEC in culture. IL-6 activity was determined using the proliferation of a B9 sensitive cell line. Results are representative of three independent experiments. Values are presented as the mean cpm of triplicate cultures (single experiment). Coefficient of variations of triplicate cultures did not exceed 10%. X axis=different dilutions of TEC supernatants. Y axis=cpm.

macrophage/myeloid differentiation, it can be postulated that this cytokine together with other hematopoietic cytokines produced by TEC (GM-CSF, M-CSF) (Haynes, 1990) can serve as growth factor for thymic macrophages and dendritic cells.

In conclusion, the system for cultivation of rat TEC *in vitro* with the addition of IFN-gamma might be useful to enhance our understanding of the functions of thymic epithelium because such an *in vitro* assay may be a better reflection of the situation existing *in vivo*.

### MATERIALS AND METHODS

### Animals

AO rats, both sexes, 5–7 weeks old, bred at the Farm for Experimental Animals (Military Medical Academy, Belgrade), were used in all experiments.

### Long-Term Cultures of Rat TEC

Thymuses were aseptically removed and minced into small fragments (2–3 mm$^3$). Fragments were then washed several times into RPMI 1640, hepes-buffered medium containing 5% fetal calf serum (FCS) and released thymocytes were discarded. All components were obtained from Flow Laboratories (Irving, Scotland). The washed explants were placed into 75 ml plastic culture flasks (Flow) containing 2 ml of bicarbonate-buffered RPMI 1640 medium, 15% FCS, 2 mM glutamine (Serva, Heidelberg, FRG), 2% gentamicin (Galenika, Belgrade, YU), 5 $\mu$l/ml insulin (Serva), and 50 nM dexamethasone (Galenika), and cultivated in a 5% $CO_2$ incubator at 37 °C. Medium was changed every third day until the cellular outgrowth formed a confluent monolayer, usually 3–4 weeks after the initiation of cultures. Under these culture conditions, predominant proliferating cells were epithelial cells growing in a mosaic-like appearance around

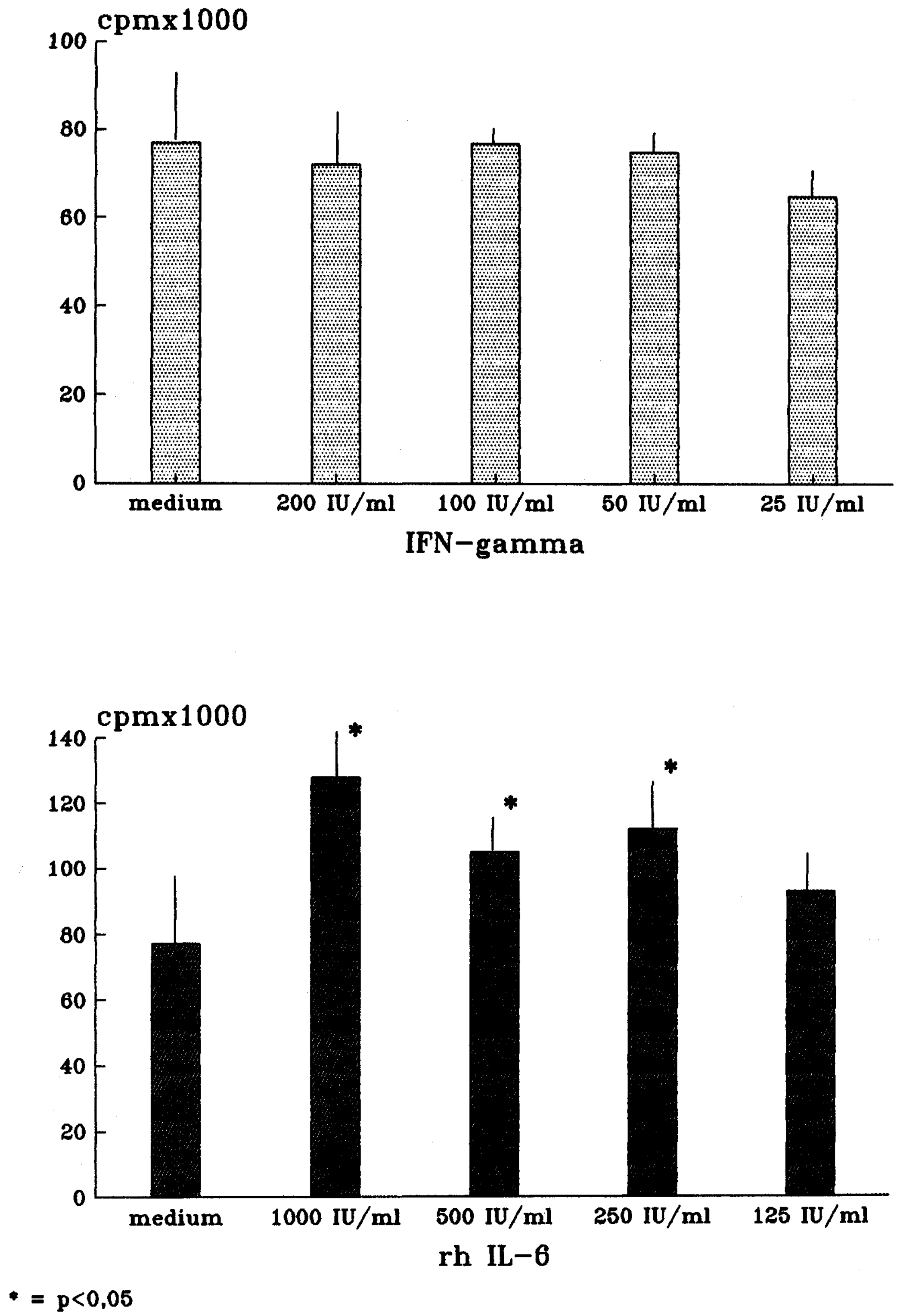

FIGURE 5. Effect of cytokines on TEC proliferation *in vitro*. TEC proliferation was determined using $^3H$ thymidine uptake as described in Materials and Methods. Results are representative of three independent experiments. Each column represents the mean of cpm of triplicate cultures ±SD (single experiment).

explants. However, cultures were contaminated with small numbers of reticular cells with fibroblastoid morphology, adipose cells, and macrophages. All these cells were easily detached using mild trypsinization with 0.03% trypsine in PBS (Torlak, Belgrade, YU) and 0.02% EDTA and vigorous pipeting. Adherent TEC were then detached using 0.08–0.10% trypsin with 0.02% EDTA and incubation at 37 °C for 10–15 min. Detached cells were washed by centrifugation and then recultivated into new flasks coated with poly-L-lysine (PLL) (Serva) in the same medium used for primary cultures with addition of 10 ng/L of epidermal growth factor (EGF) (ICN-Galenika, Belgrade, YU). TEC were successfully grown under these conditions and propagated for more than 8 months. Now they are still in culture.

### Antibodies, Cytokines, and Reagents

A large panel of monoclonal antibodies (mAbs) reactive with subsets of rat TEC were used (Table 1). R-MC series of mAbs were produced in our laboratory (Čolić et al., 1988) and HIS series of mAbs (Kampinga et al., 1987) were obtained from Dr. Kampinga (Dept. of Histology and Cell Biology, University of Groningen, The Netherlands). MAbs reactive with cytokeratin-polypeptides (CK) 8, 18, and 19, respectively, were obtained from Amersham International (Amersham, Bucks, UK). Monoclonal anti-CK antibodies reactive with several CK polypeptides (K.8.13) and CK pair 13 and 16 (K.8.12) were purchased from ICN-Galenika, and KL1 mAb reactive with CK 10 were obtained from Serotec, UK.

Recombinant human IL-6 was purchased from Genzyme (Boston, MA). Recombinant rat IFN-gamma was a generous gift from Dr. P. van der Meide (TNO, Rijswik, The Netherlands). Secondary biotinylated antibodies (goat antimouse Ig), sheep antimouse Ig coupled with fluorescein isothyocyanate (FITC), as well as streptavidin-FITC were purchased from Amersham.

### Preparation of TEC-Conditioned Medium

One long-term TEC culture passaged for 8 months was used for phenotypic analysis and electron microscopy. In addition, confluent cultures were washed and incubated with RPMI 1640, containing 10% FCS and different doses of recombinant rat IFN-gamma (25, 50, 100, and 200 IU/ml). Supernatants were collected after 48 hr incubation and then centrifuged to remove cellular remnants. If not immediately used for cytokine assays, samples were stored at –20 °C.

### B9 Bioassay for IL-6

IL-6 activity was measured by $^3$H thymidine incorporation in the IL-6-dependent murine hybridoma cell line B9 (Aarden et al., 1987) kindly provided by Dr. L. A. Aarden (Central Laboratory of Netherland, Amsterdam).

B9 cells were seeded $0.5\times10^4$ cells per culture and different dilutions of test samples or recombinant human IL-6 were added. Cultures were incubated 72 hr at 37 °C and 5% $CO_2$, pulsed for the last 8 hr of incubation with $^3$H thymidine, harvested, and counted. Results are given in counts per minute (cpm). Specificity of IL-6-dependent proliferation was previously confirmed using neutralizing rabbit antihuman IL-6 antibody (Čolić et al., manuscript submitted).

### Immunohistochemical Staining

Cytospins of trypsinized TEC cultures or monolayers of TEC grown on coverslips coated with PLL were air dried overnight and fixed in acetone for 10 min. Slides were then incubated overnight at 4 °C with appropriate dilutions of mAbs followed by washing in TRIS-buffered saline (TBS), pH 7.6, incubation for 30 min with antimouse biotinylated antibody (dilution 1:100) in TBS containing 2% normal rat serum, washing and subsequent incubation with streptavidin-FITC (1:100) for 30 min. Finally, slides were mounted in glycerol and observed under a fluorescence microscope, Univar III.

Specificity of staining was checked using irrelevant mouse mAbs of the same isotype (produced in our laboratory).

### Electron Microscopy

Cultures were trypsinized with 0.04% trypsin/EDTA allowing cell detachment in large clusters. Cells were then centrifuged, fixed with 1% glutaraldehyde in 0.1 M Na-cacodylate buffer, pH 7.4, and postfixed in 2% osmium tetroxide. After dehydration, embedding in Epon, sectioning and contrasting with lead citrate and uranyl-acetate, the specimens were examined in a Philips EM 200 electron microscope.

### Flow Cytometry

Single-cell suspensions of trypsinized TEC cultures (0.10% trypsine/0.02% EDTA) were prepared and adjusted to the concentration of $1\times10^6$ cells/tube. Viability of cells was 96%. TEC were then stained with OX-6, OX-17, or OX-18 mAbs, washed in PBS containing 0.01% sodium azide and 2% FCS, followed by rabbit anti-Ig FITC antibody. Negative controls consisted of an irrelevant mouse IgG1 antibody produced in our laboratory. Cells were analyzed on an EPICS-CS flow cytometer (Coulter, FRG).

### TEC Proliferation Assay

Trypsinized TEC were plated into 96-well flat-bottom plates (Flow) at a density of $5\times10^3$ cells/well in 0.01 ml of culture medium used for long-term growth of TEC as previously described. After 24 hr, adherent cells were washed three times with PBS and then culture medium consisting of RPMI 1640 medium with 10% FCS and devoid of EGF, hydrocortisone, and insulin was added. TEC were cultivated for further 24 hr, when different doses of either IFN-gamma, IL-6, or medium alone were added to the wells in triplicate. After 24 hr, TEC were pulsed with 1 μCi of $^3$H thymidine/per well (5 mCi/mmol, specific activity) (Amersham). At 48 hr, the plates were washed with PBS and cells were trypsinized using 0.1% trypsin/0.02% EDTA for 15 min. The radioactivity of the samples was measured and the results were expressed as the means of counts per minute (cpm) of triplicate.

### ACKNOWLEDGMENTS

The authors wish to thank Dr. P. van der Meide for the gift of recombinant rat INF-gamma, Dr. L. A. Aarden for the B9 cell line, and Dr. I. Kampinga for the HIS series of mAbs. We also thank Dr. N. Stojanović for technical assistance and Dr. D. Lilić for critical evaluation of the manuscript.

*(Received May 14, 1991)*

*(Accepted September 25, 1991)*

### REFERENCES

Aarden A.L., De Groot E.R., Schaap O.L., and Lansdorp P.M. (1987). Production of hybridoma growth factor by human monocytes. Eur. J. Immunol. **17:** 1411–1416.

Berrih S.F., Arenzana-Seisdedos F., Cohen S., Devos R., Charron D., and Virelizier J. (1985). Interferon-gamma modulates HLA class II antigen expression on cultured thymic epithelial cells. J. Immunol. **135:** 1165–1171.

Čolić M., Jovanović S., Mitrović S., and Dujić A. (1989). Immunohistochemical identification of six cytokeratin-defined subsets of the rat thymic epithelial cells. Thymus **13:** 175–185.

Čolić M., Matanović D., Hegediš L., and Dujić A. (1988). Immunohistochemical characterization of rat thymic non-lymphoid cells. I. Epithelial and mesenchymal components defined by monoclonal antibodies. Immunology **65:** 277–284.

Ehmann U., Shiurba R.A., and Peterson W.D., Jr. (1986). Long-term proliferation of mouse thymic epithelial cells in culture. In Vitro **22:** 738–749.

Eshel I., Savion N., and Shocam J. (1990). Analysis of thymic stromal cell subpopulations grown *in vitro* on extracellular matrix in defined medium. I. Growth conditions and morphology of murine thymic epithelial and mesenchymal cells. J. Immunol. **144:** 1554–1562.

Farr A.G., Eisenhardt J., and Anderson S.K. (1986). Isolation of murine thymic epithelium and improved method for its propagation *in vitro*. Anat. Rec. **216:** 85–94.

Farr A.G., Hosier S., Braddy S.C., Anderson S.K., Eisenhardt D.J., Yan Z.J., and Robles C.P. (1989). Medullary epithelial cell lines from murine thymus constitutively secrete IL-1 and hematopoietic growth factors and express class II antigens in response to recombinant interferon-gamma. Cell. Immunol. **119:** 427–435.

Galy A.H.M., Hadden E.M., Tourine J.-F., and Haden J.W. (1989). Effect of cytokines on human thymic epithelial cells in culture: IL-1 induce thymic epithelial cell proliferation and change in morphology. Cell. Immunol. **124:** 13–20.

Haynes B.F. (1990). Human thymic epithelium and T cell development: Current issues and future direction. Thymus **16:** 143–157.

Howells G., Pham P., Taylor D., Foxwell B., and Feldmann M. (1991). Interleukin 4 induces interleukin 6 production by endothelial cells: synergy with interferon-gamma. Eur. J. Immunol. **21:** 97–101.

Janson J.M., and Janeway C.A. (1984). *in vitro* cultivation of nonlymphoid thymic cells: Morphological and immunological characterization. Clin. Immunol. Immunopathol. **30:** 214–226.

Kampinga J., Kroese F.G.M., Duijvestijn A.M., Murawska M.B., Pol G.H., and Nieuwenhuis P. (1987). The rat thymus microenvironment: Subsets at rat thymic epithelial cells defined by monoclonal antibodies. Transpl. Proc. **19:** 3137–3139.

Kohase M., Henriksen-DeStefano D., May L.T., Vilcek T., and Sehgal P.B. (1986). Induction of beta2-interferon by tumor necrosis factor: A homeostatic mechanism in the control of cell proliferation. Cell **45:** 659–666.

Le T.P., Lazorick S., Whichard L.P., Yang Y.-C., Clark S.C., Haynes B.F., and Singer K.H. (1990). Human thymic epithelial cells produce IL-6 granulocyte-monocyte-CSF and leukemia inhibitory factor. J. Immunol. **145:** 3310–3315.

Le T.P., Tuck D.T., Dinarello A.C., Haynes B.F., and Singer K.H. (1987). Human thymic epithelial cells produce interleukin 1. J. Immunol. **138:** 2520–2526.

Lobach D.F., and Haynes B.F. (1988). Ontogeny of the human thymus during fetal development. J. Clin. Immunol. **7:** 81–97.

Murray K., Suda T., Wrighton N., Lee F., and Zlotnick A. (1989). IL-7 is a growth and maintenance factor for mature and immature thymocyte subsets. Int. Immunol. **1:** 526–532.

Nieburgs A.C., Picciano P.T., Korn J.H., McCalister T., Allred C., and Cohen S. (1985). *in vitro* growth and maintenance of two morphologically distinct populations of thymic epithelial cells. Cell. Immunol. **90:** 439–450.

Ogawa M., and Clark C.S. (1988). Synergistic interaction between interleukin-6 and interleukin-3 support of stem cell proliferation in culture. Blood Cells **14:** 329–337.

Osculati F., Balercia G., and Mathe G. (1988). Human thymic epithelium in culture: An experimental model for the study of thymic microenvironment. Biomed. Pharmacoter. **42:** 395–407.

Paul S.R., Bennett F., Calvetti J.A., Kelleher K., Wood C.R., O'Hara R.M., Leary A.C., Sibley B., Clark S.C., Williams D.A., and Yang Y.-C., (1990). Molecular cloning of a cDNA encoding interleukin 11, a stromal cell-derived lymphopoietic and hematopoietic cytokine. Proc. Natl. Acad. Sci. USA **87:** 7512–7516.

Piltch A., Naylor F., and Hayashi J. (1988). A cloned rat thymic epithelial cell line established from serum-free selective culture. In Vitro **24:** 289–299.

Potworowski E.F., Turcotte F., Beauchemin C., Hugo P., and Zelechowska M.G. (1986). Establishment and characterization of a thymic medullary epithelial cell clone. In Vitro **22:** 557–565.

Ransom J., Fischer M., Mercer L., and Zlotnik A. (1987). Lymphokine-mediated induction of antigen-presenting ability in thymic stromal cells. J. Immunol. **139:** 2620–2628.

Sehgal P.B. (1990). Interleukin 6: Molecular pathophysiology. J. Investig. Dermatology **94 (Supp.):** 25–33.

Small M., Bar-Nea L., and Aronson M. (1984). Cultures of thymic epithelial cells from mice and age related studies on the growing cells. Eur. J. Immunol. **14:** 936–942.

Small M., van Ewijk W., Gown A.M., and Rouse R.V. (1989). Identification of subpopulations of mouse thymic epithelial cells in culture. Immunology **68:** 371–377.

Sprent J., Lo D., Gao E.-K., and Ron Y. (1988). T cell selection in the thymus. Immunol. Rev. **101:** 173–190.

Suda T., Murray R., Guidas C., and Zlotnik A. (1990). Growth-promoting activity of IL-1 alfa, IL-6 and tumor necrosis factor-alfa in combination with IL-2, IL-4 or IL-7 on murine thymocytes. Differential effect on CD4/CD8 subsets and on CD3+/CD3– double-negative thymocytes. J. Immunol. **144:** 3039–3045.

Sun T.T., Bonitz P., and Burns W.H. (1984). Cell culture of mammalian thymic epithelial cells: Growth, structural and antigenic properties. Cell. Immunol. **83:** 1–13.

Van Ewijk W. (1988). Cell surface topography of thymic microenvironments. Lab. Investig. **59:** 579–590.

Van Snick J. (1990). Interleukin-6: An overview. Annu. Rev. Immunol. **8:** 253–278.

